# Letter from the Editor in Chief

**DOI:** 10.19102/icrm.2021.120406

**Published:** 2021-04-15

**Authors:** Moussa Mansour


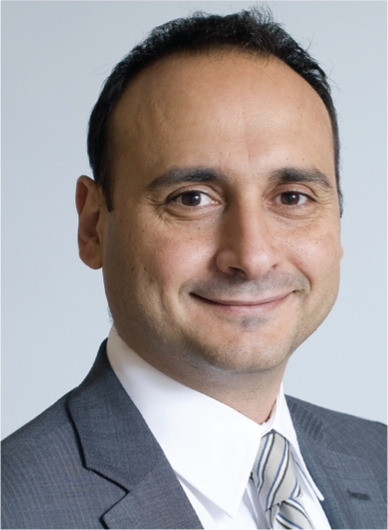


Dear Readers,

Radiofrequency (RF) energy is a powerful tool that allows for the rapid and durable inactivation of abnormal foci of electrical activity for numerous arrhythmias. In particular, it has helped to accelerate the use of catheter ablation for atrial fibrillation (AF), which now accounts for the majority of ablation procedures performed. Despite the benefits of RF energy, however, its use is associated with a real—albeit small—risk of complications, including pulmonary vein stenosis, injury to the esophagus, and phrenic nerve injury, all due to the thermal effect of RF energy.

Pulsed-field ablation (PFA) consists of applying brief, high-intensity electric shocks that result in irreversible electroporation and subsequent cell death, thus offering the promise not only of creating lesions without heating or freezing but also of being selective for cardiac cells. Numerous preclinical studies have demonstrated its ability to spare the esophagus and phrenic nerve during ablation,^1^ and first-in-human research showed that it has no effect on the diameter of the pulmonary veins following ablation.^2^ Its short-term efficacy has also been demonstrated with a high rate of acute pulmonary vein isolation, which was also proven to be durable based on remapping studies.^3^ Moreover, PFA appears to be an efficient modality, with very short procedure times.^3^ While data regarding its long-term efficacy are not yet available, randomized multicenter clinical studies are currently comparing its one-year outcomes with those of conventional ablation methods.

Assuming that the impressive short-term results of PFA will translate into long-term efficacy, I believe that PFA will be a “disruptive technology”—a term invented by Clayton M. Christensen, a professor at Harvard Business School, to refer to a beneficial technology that “displaces a well-established product or technology, creating a new industry or market”—in the field of cardiac electrophysiology. It is likely that pulmonary vein isolation will be simplified, similar to the ablation of supraventricular tachycardia, requiring less infrastructure and skill, resulting in a huge benefit to the millions of people with AF.

Best regards and I hope that you find the content of this issue of *The Journal of Innovations in Cardiac Rhythm Management* beneficial and educational.

Sincerely,


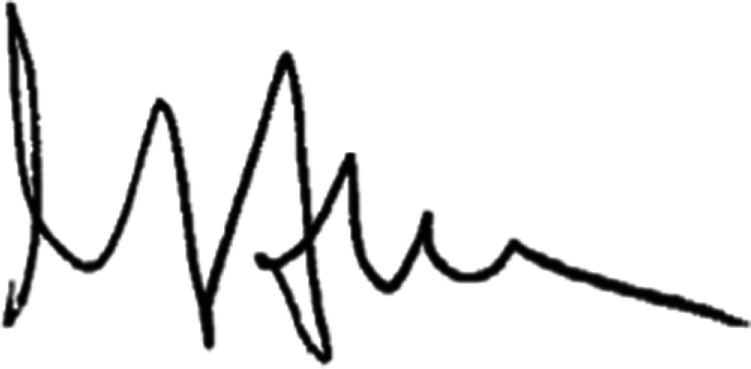


Moussa Mansour, md, fhrs, facc

Editor in Chief

The Journal of Innovations in Cardiac Rhythm Management

MMansour@InnovationsInCRM.com

Director, Atrial Fibrillation Program

Jeremy Ruskin and Dan Starks Endowed Chair in Cardiology

Massachusetts General Hospital

Boston, MA 02114

## References

[1] Koruth JS, Kuroki K, Kawamura I (2020). Focal pulsed field ablation for pulmonary vein isolation and linear atrial lesions: a preclinical assessment of safety and durability. Circ Arrhythm Electrophysiol.

[2] Kuroki K, Whang W, Eggert C (2020). Ostial dimensional changes after pulmonary vein isolation: Pulsed field ablation vs radiofrequency ablation. Heart Rhythm.

[3] Reddy VY, Anic A, Koruth J (2020). Pulsed field ablation in patients with persistent atrial fibrillation. J Am Coll Cardiol.

